# Supplementary material for: Modeling glioblastoma heterogeneity as a dynamic network of cell states
Source: Mol Syst Biol. 2021 Sep 16;17(9):e10105. doi: 10.15252/msb.202010105 (PMC8444284; doi:10.15252/msb.202010105)
Supplement: Supplementary file 6 — Source Data for Figure 5 [file MSB-17-e10105-s004.zip › Figure5A_sourcedata/GSEA_3071/hallmarks_stateI.GseaPreranked.1623742531165/HALLMARK_DNA_REPAIR.html]

Details for gene set HALLMARK\_DNA\_REPAIR[GSEA]

|  || Dataset | state13071 |
| Phenotype | NoPhenotypeAvailable |
| Upregulated in class | na\_neg |
| GeneSet | HALLMARK\_DNA\_REPAIR |
| Enrichment Score (ES) | -0.49837846 |
| Normalized Enrichment Score (NES) | -2.2432365 |
| Nominal p-value | 0.0 |
| FDR q-value | 4.3362996E-4 |
| FWER p-Value | 0.003 |
Table: GSEA Results Summary

  

Fig 1: Enrichment plot: HALLMARK\_DNA\_REPAIR      
 Profile of the Running ES Score & Positions of GeneSet Members on the Rank Ordered List

  

| PROBE | GENE SYMBOL | GENE\_TITLE | RANK IN GENE LIST | RANK METRIC SCORE | RUNNING ES | CORE ENRICHMENT || 1 | DAD1 |  |  | 486 | 0.524 | -0.1181 | No |
| 2 | POLD4 |  |  | 810 | 0.378 | -0.1953 | No |
| 3 | ITPA |  |  | 1010 | 0.326 | -0.2389 | No |
| 4 | NUDT9 |  |  | 1260 | 0.275 | -0.2991 | No |
| 5 | USP11 |  |  | 1522 | -0.260 | -0.3634 | No |
| 6 | NELFCD |  |  | 1724 | -0.283 | -0.4095 | No |
| 7 | RAD51 |  |  | 1845 | -0.299 | -0.4314 | No |
| 8 | POLR2E |  |  | 1941 | -0.312 | -0.4457 | No |
| 9 | DGCR8 |  |  | 1991 | -0.317 | -0.4464 | No |
| 10 | PDE4B |  |  | 2132 | -0.339 | -0.4725 | No |
| 11 | NCBP2 |  |  | 2204 | -0.353 | -0.4780 | No |
| 12 | PNP |  |  | 2223 | -0.358 | -0.4681 | No |
| 13 | GTF2A2 |  |  | 2329 | -0.379 | -0.4824 | Yes |
| 14 | VPS37B |  |  | 2363 | -0.386 | -0.4756 | Yes |
| 15 | UMPS |  |  | 2368 | -0.387 | -0.4603 | Yes |
| 16 | POLA1 |  |  | 2376 | -0.388 | -0.4460 | Yes |
| 17 | ERCC1 |  |  | 2397 | -0.392 | -0.4351 | Yes |
| 18 | IMPDH2 |  |  | 2417 | -0.396 | -0.4239 | Yes |
| 19 | POLA2 |  |  | 2452 | -0.401 | -0.4167 | Yes |
| 20 | HPRT1 |  |  | 2454 | -0.402 | -0.4000 | Yes |
| 21 | POLR2D |  |  | 2510 | -0.416 | -0.3983 | Yes |
| 22 | NME4 |  |  | 2518 | -0.418 | -0.3826 | Yes |
| 23 | SSRP1 |  |  | 2580 | -0.434 | -0.3818 | Yes |
| 24 | DUT |  |  | 2640 | -0.450 | -0.3798 | Yes |
| 25 | NME1 |  |  | 2669 | -0.463 | -0.3683 | Yes |
| 26 | TAF10 |  |  | 2689 | -0.470 | -0.3539 | Yes |
| 27 | AK3 |  |  | 2716 | -0.475 | -0.3413 | Yes |
| 28 | RPA3 |  |  | 2730 | -0.480 | -0.3247 | Yes |
| 29 | EIF1B |  |  | 2747 | -0.485 | -0.3088 | Yes |
| 30 | COX17 |  |  | 2786 | -0.498 | -0.2987 | Yes |
| 31 | ARL6IP1 |  |  | 2823 | -0.510 | -0.2875 | Yes |
| 32 | LIG1 |  |  | 2848 | -0.520 | -0.2725 | Yes |
| 33 | RFC5 |  |  | 2862 | -0.526 | -0.2540 | Yes |
| 34 | POLR2I |  |  | 2887 | -0.534 | -0.2383 | Yes |
| 35 | ALYREF |  |  | 3068 | -0.649 | -0.2628 | Yes |
| 36 | SAC3D1 |  |  | 3073 | -0.655 | -0.2363 | Yes |
| 37 | GPX4 |  |  | 3103 | -0.679 | -0.2159 | Yes |
| 38 | RFC3 |  |  | 3108 | -0.683 | -0.1882 | Yes |
| 39 | POLD3 |  |  | 3122 | -0.695 | -0.1625 | Yes |
| 40 | RFC4 |  |  | 3129 | -0.701 | -0.1346 | Yes |
| 41 | PRIM1 |  |  | 3160 | -0.739 | -0.1120 | Yes |
| 42 | RFC2 |  |  | 3245 | -0.839 | -0.1008 | Yes |
| 43 | FEN1 |  |  | 3278 | -0.890 | -0.0723 | Yes |
| 44 | ZWINT |  |  | 3296 | -0.925 | -0.0381 | Yes |
| 45 | PCNA |  |  | 3319 | -0.963 | -0.0037 | Yes |
| 46 | TYMS |  |  | 3436 | -1.391 | 0.0216 | Yes |
Table: GSEA details [plain text format]

  

Fig 2: HALLMARK\_DNA\_REPAIR: Random ES distribution      
 Gene set null distribution of ES for **HALLMARK\_DNA\_REPAIR**
